# Effectiveness of a Self-Esteem Enhancement Intervention Integrated into Standard CBT Protocol for Improving Quality of Life in Patients with Colorectal Cancer

**DOI:** 10.3390/ejihpe15040042

**Published:** 2025-03-22

**Authors:** Lavinia Alina Rat, Timea Claudia Ghitea, Adrian Marius Maghiar

**Affiliations:** 1 Doctoral School, Faculty of Medicine and Pharmacy, University of Oradea, 410068 Oradea, Romania; lavirat@yahoo.com; 2 Pharmacy Department, Faculty of Medicine and Pharmacy, University of Oradea, 410068 Oradea, Romania; amaghiar@gmail.com; 3 Medicine Department, Faculty of Medicine and Pharmacy, University of Oradea, 410068 Oradea, Romania

**Keywords:** cognitive behavioral therapy, colorectal cancer, quality of life, self-esteem, psychological intervention

## Abstract

Cognitive behavioral therapy (CBT) has proven effective in addressing the psychological and emotional challenges of cancer. This study evaluated the impact of a self-esteem enhancement intervention integrated into a standard CBT protocol on the quality of life and self-esteem of women with colorectal cancer. Conducted at Spitalul Județean Clinic Bihor (Romania) between August 2020 and March 2024, the study included 67 women aged 32 to 66 years undergoing chemotherapy or combined chemotherapy and radiotherapy. This study aimed to evaluate the impact of a self-esteem enhancement intervention integrated into a standard CBT protocol on the quality of life and self-esteem of women with colorectal cancer. Participants were assigned to a CBT group, receiving eight weekly sessions based on the Simonton Program, or a control group on a psychotherapy waiting list. Quality of life was assessed using the EORTC QLQ-C30 questionnaire, and self-esteem was measured through actual and ideal self-perceptions. A repeated measures ANOVA analyzed changes in both outcomes. Results showed a significant improvement in quality of life (F = 6.33, df = 1, 65, *p* < 0.05) and self-esteem (F = 4.46, df = 1, 65, *p* < 0.05) in the CBT group, whereas no improvements were observed in the control group. Self-esteem was enhanced through reduced discrepancies between actual and ideal self-perceptions, especially in cognitive and emotional dimensions. Physical functioning improved but was less pronounced, influenced by disease progression and treatment stage. No significant changes were observed in social functioning, suggesting that longer-term interventions may be needed. These findings highlight CBT as a valuable complementary intervention in oncology care, supporting its integration into standard treatment to enhance patients’ psychological well-being and quality of life.

## 1. Introduction

Self-esteem represents an indicator of personal acceptance, tolerance, and satisfaction with oneself, stemming from the affective assessment of the attributes an individual associates with themselves (Niveau et al., 2021). Numerous theoretical frameworks have redefined self-esteem. Based on these perspectives, self-esteem plays a crucial role in psychological resilience and emotional adjustment in patients with chronic diseases, including cancer (Cast & Burke, 2002). Low self-esteem has been associated with higher psychological distress and poorer treatment adherence (Wojtyna et al., 2023). For example, correlational analyses reveal that high self-esteem encourages motivation, persistence amid adversities, greater goal-oriented effort, and health-enhancing behaviors (Baumeister et al., 2003). Conversely, low self-esteem correlates with frequent physical symptoms and is a common transdiagnostic symptom of many mental disorders (Silverstone & Salsali, 2003).

Cognitive behavioral therapy (CBT) is a structured psychological intervention that targets maladaptive thoughts and behaviors to enhance coping mechanisms (Schmeichel et al., 2009). In oncology settings, CBT has been shown to reduce anxiety, improve emotional resilience, and enhance quality of life in cancer patients.

Notably, extensive empirical findings support the idea that low self-esteem constitutes a risk factor for depression. Individuals with low self-esteem exhibit greater vulnerability to the psychological effects of negative experiences (Orth et al., 2014). Some studies identified that participants with low self-esteem experienced more intense effects when encountering adverse events compared to individuals with high self-esteem (Samuels & Dale, 2022). Moreover, negative events tend to affect all evaluations—relevant or unrelated to the situation—more significantly in individuals with low self-esteem than in those with higher levels (Josephs et al., 2003). Consequently, self-esteem may predict psychological outcomes following life-changing events (Reitz, 2022).

Terror management theory views self-esteem as a protective mechanism against existential fear related to mortality (Schmeichel et al., 2009). High self-esteem may help individuals mitigate anxiety resulting from awareness of inevitable mortality by fostering inclusion and value within society (Gyimes & Valentini, 2023). Maintaining high self-esteem in patients could enhance resilience against life-threatening illnesses like cancer, which often heightens existential anxiety. Nevertheless, findings about the effects of self-esteem should be carefully considered due to limitations associated with research in this domain (e.g., subjective measurements, confounding variables) (Sáez Rodríguez et al., 2024). Studies have consistently highlighted relationships between self-esteem levels and coping mechanisms in cancer patients.

In 2018, it was demonstrated that acceptance of cancer-related limitations correlated with greater self-esteem. Joaquín-Mingorance et al. (2019) identified that adaptive coping strategies (e.g., positive reframing, emotional support, active coping, planning, and acceptance) in breast cancer patients were positively associated with self-esteem (Joaquín-Mingorance et al., 2019). Social support is also strongly linked with self-esteem, aligning with sociometer theory, which defines self-esteem as an indicator of social inclusion. The connection between social support and self-esteem has been investigated in cancer patients and is crucial. Positive relationships with others appear more strongly associated with self-esteem in women with cancer compared to healthy women of similar age (Holmstrom & Lim, 2023; Kleiman & Riskind, 2013).

The interconnections between self-esteem, coping, and social support suggest potential roles for self-esteem in depressive disorders among cancer patients (Gabrić, 2024). Psychological processes explaining these relationships propose that individuals with lower self-esteem tend to ruminate on negative aspects of their self-concept, increasing depressive tendencies (Sun et al., 2023). Additionally, such individuals may excessively seek reassurance from others or interpret negative social feedback to align with their negative self-concept (Sebri et al., 2021).

This study aimed to evaluate the impact of CBT combined with specific interventions targeting self-esteem on improving the quality of life in women diagnosed with colorectal cancer. It sought to investigate the effectiveness of these interventions in enhancing the cognitive, emotional, and social dimensions of self-esteem while reducing physical and psychological symptoms linked to cancer and its treatment.

## 2. Materials and Methods

This clinical trial was conducted at “Spitalul Clinic Județean Bihor”, a tertiary oncology care center in Oradea, Romania. Participants were recruited from outpatient oncology clinics and radiotherapy units within the healthcare network of “Rat Lavinia” Psychology Medical Office, Oradea. Patient enrollment occurred between August 2020 and March 2024. Participants were recruited from the oncology outpatient department and radiotherapy units of “Spitalul Județean Clinic Bihor”, a regional referral center for cancer treatment.

### 2.1. Participants and Study Design

This controlled clinical trial included 67 women diagnosed with colorectal cancer who were undergoing chemotherapy or a combination of chemotherapy and radiotherapy. The study design involved two groups with assessments conducted at two points: baseline (pre-intervention) and post-intervention.

The experimental group (*n* = 35) participated in group cognitive behavioral therapy (CBT) sessions structured on the Simonton Program, a well-established intervention for oncological patients. The control group (*n* = 32) comprised women on a waiting list for psychotherapy.

### 2.2. Inclusion/Exclusion Criteria

Participation was voluntary, with written informed consent obtained from all participants. Inclusion criteria were absence of significant cognitive impairment, no active psychotic disorders, no history of mental illness, and no prior professional psychotherapy experience.

Inclusion criteria were women aged 32–66 years, diagnosed with colorectal cancer (Stage I–III), undergoing chemotherapy or combined chemotherapy and radiotherapy, with no prior professional psychotherapy experience.

Exclusion criteria were women with significant cognitive impairment, active psychotic disorders, or advanced-stage metastatic cancer.

The evaluations for cognitive impairment were conducted by a psychiatrist, but due to retrospective data collection, specific assessment tools were not documented.

### 2.3. Methodology

This study employed a randomized controlled trial (RCT) design, which allows for strong causal inferences regarding the effectiveness of CBT in improving self-esteem and quality of life in colorectal cancer patients undergoing chemotherapy.

### 2.4. Data Collection Methods

Data were collected using validated self-report measures of self-esteem (Rosenberg Self-Esteem Scale) and quality of life (The European Organization for Research and Treatment of Cancer QLQ-C30). Both instruments are widely used and have established reliability and validity in cancer populations.

To evaluate quality of life and self-esteem, validated instruments with high sensitivity to changes over time were employed:

EORTC QLQ-C30 Quality of Life Questionnaire

The EORTC QLQ-C30 is a standardized tool widely used among cancer patients. It assesses quality of life across physical, emotional, cognitive, and social dimensions, including daily functioning and symptom intensity (e.g., fatigue, pain, nausea). High scores in functional and global assessments indicate better quality of life, while high symptom scores reflect increased symptom severity and potential condition deterioration (Groenvold et al., 1997).

Self-Esteem Scale Developed by R. Cibor

Self-esteem was assessed using a specially designed scale with two components: “How are you now?” (current self-perception) and “How would you like to be?” (aspirations and ideals). Each version comprises 18 items in six categories: physical, mental, emotional, interpersonal, action, and social traits. Responses are measured on a seven-point Likert scale, with higher scores denoting greater intensity of the assessed factors. The difference between the “real” and “ideal” self scores represents self-esteem, where smaller discrepancies indicate higher self-esteem (Zhang et al., 2023).

Self-Esteem Indicators

The “real self”, measured by Self-Esteem Scale 1, reflects the current perception of identity, while the “ideal self”, assessed by Self-Esteem Scale 2, represents aspirations and goals. The score discrepancy quantifies the gap between current perceptions and ideals, with smaller gaps indicating greater self-esteem. Prior studies have validated the scale’s reliability and internal consistency, particularly for assessing self-esteem in oncological contexts, such as in women post-mastectomy.

Sensitivity of the Instruments

Both the EORTC QLQ-C30 and the Self-Esteem Scale are validated tools sensitive to temporal changes. Their applicability to repeated measurements ensures they effectively capture the psychological intervention’s impact on patients’ quality of life and self-esteem.

### 2.5. Intervention Method

The EORTC QLQ-C30 (quality of life) and the Self-Esteem Scale (Bochenek-Cibor et al., 2021) were administered through structured interviews conducted by trained psychologists at baseline and after the intervention.

Assessments were conducted at two time points: baseline (before the intervention) and post-intervention (at eight weeks).

The CBT intervention was designed to address the psychological and emotional needs of women diagnosed with colorectal cancer. Its objectives included reducing psychological distress and enhancing self-esteem, ultimately aiming to improve patients’ quality of life.

The therapy focused on identifying and modifying dysfunctional automatic thoughts related to the disease and treatment, such as “Cancer is destroying my life” or “I cannot cope with the treatment”. Follow-up assessments were conducted at baseline (pre-intervention) and post-intervention (eight weeks after the start of therapy). By replacing negative beliefs with adaptive perspectives, the intervention aimed to improve emotional well-being, encourage positive behaviors, and enhance coping skills.

The intervention consisted of eight weekly group sessions, each lasting two hours, conducted in a specialized clinical setting. Groups included 10–12 participants, and attendance was voluntary. Participants could bring support partners (spouses, friends, or family members); nine participants opted to attend without a support partner. The therapy was delivered by psychologists trained in cognitive-behavioral interventions.

The therapeutic program followed a CBT framework adapted from the Simonton Program, with a particular emphasis on boosting self-esteem and emotional regulation. Techniques included guided imagery to foster positive thinking and hope, along with personalized strategies to help patients manage stress and negative emotions. The intervention also aimed to strengthen social connections and leverage support networks. A focus on addressing fear of death and cultivating a positive outlook on life, even in the context of cancer, was central to the intervention. This tailored approach considered the unique challenges colorectal cancer poses to self-esteem.

### 2.6. Statistical Analysis

**Figure 1:**
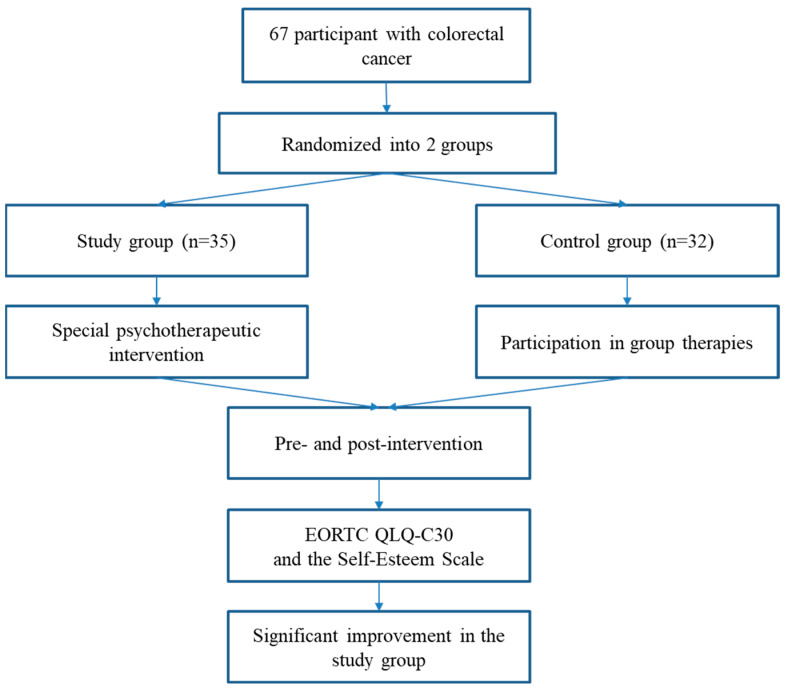
Flowchart.

Data analysis was conducted using the Statistical Product and Service Solutions (SPSS, version 20; IBM, Armonk, NY, USA) software. Descriptive statistics were used to summarize demographic variables, procedure frequency, and cost data collected from practice records at two selected time points. The assessment was performed separately for the two study groups to identify significant trends and relevant differences between them, presented in the flowchart (Figure 1).

Descriptive statistics: Means, standard deviations, medians, interquartile ranges, and percentages were calculated to summarize continuous and categorical variables.

Comparative analysis between groups: Student’s *t*-test (for independent samples) was used to compare continuous variables between the study groups. Paired *t*-test was applied to compare within-group differences between baseline and post-intervention values. The chi-square test (χ^2^) was used to analyze categorical variables and assess group differences in proportions.

Correlation analysis: The Bravais–Pearson correlation coefficient was used to measure the relationship between two continuous variables, while Spearman’s rank correlation was applied for ordinal data.

Analysis of variance (ANOVA): A repeated measures ANOVA was conducted to assess within-group and between-group differences over time.

Post hoc analysis: The Bonferroni correction was applied for multiple comparisons in post hoc subgroup analyses to control for type I error.

Effect size calculation: Cohen’s d was used to estimate the effect size for mean differences between groups, while partial eta squared (η^2^) was calculated for ANOVA models.

Regression analysis: Multiple linear regression models were used to explore potential predictors of quality of life and self-esteem improvements.

Significance thresholds: A significance level of *p* < 0.05 was considered statistically significant, while *p* < 0.01 indicated high-level statistical significance.

Power analysis: For a two-tailed alpha of 0.05 and an assumed correlation of 0.6 between pre- and post-intervention assessments, a sample size of N = 128 provided at least 80% statistical power.

Covariates: The analyses controlled for potential covariates, including age, cancer stage, and treatment modality

### 2.7. Informed Consent and Ethical Considerations

Ethical considerations were rigorously adhered to throughout the study. Informed consent was obtained from all participants, and they were made fully aware of their right to withdraw from the study at any time without penalty. Participants were also assured of the confidentiality of their data, and all study procedures were approved by the institutional review board (IRB). There were no reported ethical concerns or adverse events related to the intervention.

## 3. Results

### 3.1. Characteristics of the Studied Groups

The two groups of participating women were comparable in marital status, educational level, and age, ensuring homogeneity. Participants ranged in age from 32 to 66 years, with the majority cohabiting with a partner and possessing a professional level of education (Table 1). The age variable was excluded from further analysis due to the absence of significant correlations with the psychological variables assessed.

A repeated measure analysis of variance (ANOVA) was conducted to evaluate the impact of cognitive behavioral therapy (CBT) on participants’ global quality of life and general self-esteem. The analysis demonstrated significant improvements in these outcomes.

Participation in CBT resulted in a marked enhancement in quality of life scores following the eight-week intervention compared to baseline measurements. Women in the experimental group exhibited a substantial increase in self-acceptance, whereas those in the control group, who did not receive the therapeutic intervention, showed no significant changes in self-acceptance or quality of life.

### 3.2. Analysis of the Actual and Ideal Self

The analysis of the actual and ideal self provided valuable insights into the factors contributing to changes in self-esteem. Statistical evaluation revealed significant differences between the CBT group and the control group regarding the level of the actual self measured after the therapy intervention (t = 2.25; df = 65; *p* < 0.05). However, no significant differences were observed in the ideal self between the two groups.

These results indicate that the improvement in self-esteem among participants in the CBT group was primarily driven by an enhanced evaluation of the actual self, while the perception of the ideal self remained unchanged (Table 2). This suggests that cognitive behavioral therapy positively influenced self-perception and general self-esteem, fostering greater confidence and self-acceptance among participants.

Interaction effects between therapy participation and physical functioning.

The interaction effects of therapy participation on physical functioning, including parameters such as physical activity level, perceived symptoms, general health status, and physical characteristics of self-esteem (Supplementary Table S2). Participants in the CBT group showed significant improvements in perceived symptoms and general health scores by the end of the intervention (F = 22.37 ***, F = 9.16 **, respectively). In contrast, the control group experienced a worsening of symptoms and a slight decline in general health status.

No statistically significant differences (NS) were observed between the two groups for physical activity level or the physical characteristics of self-esteem. These findings indicate that the CBT intervention had a positive impact on symptom perception and health status, although it did not significantly influence physical activity levels or the physical dimensions of self-esteem.

### 3.3. Effects of Cognitive Behavioral Therapy (CBT) on Cognitive Functioning

The effects of participating in cognitive behavioral therapy (CBT) on cognitive functioning, highlighting significant changes from baseline to post-test assessments. In the CBT group, cognitive functioning improved substantially, with scores increasing from 58.10 to 72.38. In contrast, the control group experienced a slight decline, with scores decreasing from 54.17 to 51.56.

Regarding self-esteem based on cognitive traits, the CBT group exhibited a significant reduction in the discrepancy between ideal and current self-perceptions, with scores improving from 6.03 to 3.21. The control group, however, showed only minimal changes, with scores shifting from 5.28 to 3.49.

Statistical analysis confirmed that the differences between the groups were significant, underscoring the effectiveness of CBT in enhancing cognitive functioning and self-esteem. The results were significant for cognitive functioning (F = 6.68 *, *p* < 0.05) and for self-esteem based on cognitive traits (F = 8.43 **, *p* < 0.01).

### 3.4. Interaction Effects Between Therapy Participation and Emotional Functioning

Table 3 presents the interaction effects of therapy participation on emotional functioning, including the emotional level assessed by the QLQ and the emotional traits of self-esteem. Participants in the cognitive behavioral therapy (CBT) group demonstrated significant improvements by the end of the intervention. Specifically, emotional functioning scores increased significantly (F = 6.68 *, *p* < 0.05), and discrepancies related to emotional traits of self-esteem were notably reduced (F = 8.43 **, *p* < 0.01).

In contrast, the control group did not exhibit meaningful changes in these dimensions. These findings indicate that CBT had a significant positive effect on participants’ emotional functioning and their emotional self-perception.

### 3.5. Results of the Therapeutic Intervention

Participants in the study reported the ability to set realistic expectations and recognize areas of progress compared to their pre-therapy state. Statistical analyses examined changes in the physical, emotional, cognitive, and social dimensions of functioning among women diagnosed with colorectal cancer. Cognitive behavioral therapy (CBT) positively influenced the perception of oncological symptoms, as well as the overall assessment of quality of life and health status (Table 4).

However, no significant differences were found between the groups regarding physical functioning, including fatigue levels, independence in daily activities, or the perception of physical aspects of self-esteem (e.g., physical efficiency, attractiveness, and sexual efficiency). This suggests that while participants perceived a reduction in symptoms, this alone was insufficient to improve physical self-evaluation. Additionally, the lack of significant results in this domain may be attributed to the varying stages of treatment among participants during the study.

Regarding emotional functioning, significant improvements were noted across all assessed domains. Women in the CBT group demonstrated better emotional functioning and enhanced positive self-esteem traits compared to the control group. These improvements were reflected in a more positive mood, inner calm, and cheerfulness—factors that can help mitigate the risk of depression and anxiety among cancer patients.

Participants in the CBT group also exhibited enhanced cognitive abilities, including improved concentration, memory retention, decision making, and overall cognitive functioning, compared to women who did not receive therapy

However, no significant differences were identified between the groups in social functioning. This dimension, which included assessments of quality of life related to interpersonal relationships and self-esteem categories such as social skills and group position, showed similar results in both groups. This suggests that changes in social functioning may require more time to become evident. The inherently bidirectional nature of social interactions likely affects this dimension, making it less amenable to short-term interventions within the timeframe of this study.

## 4. Discussion

The results of this study confirm the positive impact of cognitive behavioral therapy (CBT) on quality of life and self-esteem, aligning with findings in the specialized literature (de Oliveira et al., 2017; Vallabadoss et al., 2023). However, these improvements are not uniformly distributed across all dimensions analyzed, as their magnitude varies depending on the nature of the variables studied.

Significant changes in self-esteem were most evident in the cognitive and emotional dimensions. This is consistent with the design of CBT, which primarily targets maladaptive thought patterns and emotional regulation. Improvements in these areas contribute to enhanced coping mechanisms, better emotional stability, and a greater sense of self-worth. Regarding quality of life, improvements in the physical dimension were observed, albeit to a lesser extent than cognitive and emotional changes. This discrepancy is likely due to the indirect nature of CBT’s effects on the physical sphere, primarily achieved by mitigating the mental influence on somatic symptoms (Białecka-Pikul et al., 2019; Chan et al., 2021).

Changes in physical symptoms are heavily influenced by disease progression and the type of pharmacological treatment administered. While reducing the number or intensity of symptoms is vital for improving quality of life, this reduction alone does not appear to significantly impact self-esteem (Dunn et al., 2003). Disease progression and the stage of treatment seem to have a more profound effect on patients’ physical self-perception, often superseding direct psychological influences.

Nevertheless, reducing physical symptoms and enhancing cognitive and emotional functioning enable patients to regain a sense of control over their condition. This regained control fosters active problem solving and better adherence to medical treatment, a finding supported by other studies on therapeutic compliance (Trudel-Fitzgerald et al., 2018). Reduced suffering also leads to greater emotional stability and lower levels of anxiety and depression, further improving overall well-being.

These findings are consistent with the theory of cognitive adaptation to cancer proposed by S. Taylor (Taylor, 1983). According to this theory, regaining a sense of control over one’s circumstances and believing in the ability to manage the disease are essential for psychological adaptation. Research suggests that even an illusory sense of control can enhance functioning and mitigate the psychological burden of the disease (Asiedu et al., 2014). Similarly, maintaining positive self-esteem, even if somewhat unrealistic, provides a protective buffer against fear and emotional disintegration (Kashdan & Rottenberg, 2010).

In contrast to the cognitive and emotional dimensions, the study found no significant changes in the social dimension. This highlights the complexity of social functioning, which is influenced by external factors beyond the patient’s direct control. Social relationships involve bilateral dynamics that can be affected by stigma, social perceptions, and the reactions of others. For instance, women with colorectal cancer may perceive positive reactions from partners as expressions of pity or as attempts to shield them from negative emotions, complicating their social interactions (Arrieta Valero, 2019; Galli et al., 2021; Prizeman et al., 2023; Reese et al., 2017).

Despite the positive results, the intervention did not show a strong effect on physical functioning, suggesting that psychological interventions may require additional complementary strategies to address the multifaceted challenges of cancer treatment. Furthermore, the study contributes novel evidence for integrating psychological care into standard cancer treatment protocols. These findings have clear clinical implications, urging healthcare providers to incorporate mental health support, such as CBT, to enhance overall well-being and quality of life for patients undergoing cancer treatment.

### 4.1. Limitations

Despite the promising results, this study has several limitations. First, the sample was relatively small, consisting only of women diagnosed with colorectal cancer, which limits the generalizability of the findings to other cancer populations or male patients. Additionally, the study focused exclusively on individuals receiving chemotherapy or combined chemotherapy and radiotherapy, which restricts its applicability to patients receiving different treatments. The short duration of the intervention, limited to eight weeks, may not have been sufficient to observe long-term benefits, particularly in areas such as physical functioning and social interactions. Moreover, the lack of follow-up data means that the sustainability of the improvements remains unknown. Lastly, the reliance on self-reported measures for assessing outcomes introduces the potential for reporting biases.

The retrospective nature of our study did not allow for the collection of data regarding the time elapsed since participants’ colorectal cancer (CRC) diagnoses. This omission introduces variability in disease duration among subjects, which may act as an unmeasured confounding factor potentially influencing self-esteem levels. The existing literature indicates that the duration of illness can significantly impact psychological outcomes in cancer patients. For instance, a study highlighted that CRC patients experience varying levels of anxiety and depression at different stages post-diagnosis, suggesting that time since diagnosis plays a crucial role in mental health outcomes (Mosher et al., 2016).

The absence of precise data on disease duration in our analysis limits our ability to control for its potential confounding effects. This limitation underscores the need for future prospective studies to systematically collect comprehensive temporal data from the point of diagnosis onward. Such data would enable a more nuanced understanding of how disease duration influences psychological outcomes, including self-esteem, among CRC patients. By accounting for this variable, future research can better isolate the effects of interventions aimed at enhancing self-esteem and quality of life in this population.

One potential limitation of the study is the possibility of selection bias. Although participants were randomly assigned to the intervention and control groups, those who volunteered for the study may have had higher baseline levels of motivation or interest in psychological interventions, which could have influenced their responses. Additionally, there were differences in baseline levels of physical health between groups, which could have confounded the results. While we controlled for these differences using ANCOVA, unmeasured confounders, such as social support or coping strategies, might have influenced the outcomes.

While the findings of this study highlight the effectiveness of CBT in enhancing quality of life and self-esteem among women with colorectal cancer, the generalizability of the results is constrained by the characteristics of the sample. The participants were exclusively women, limiting the applicability of the findings to male cancer patients or those with other types of cancer. Furthermore, the relatively homogenous demographic profile of the sample, including marital status, education level, and age range, restricts the external validity of the study to broader and more diverse populations. However, the relatively small sample size limits the power of the study and its generalizability to broader populations. The use of self-report measures, though validated, may have introduced response biases, which could affect the reliability of the findings. Future studies could include objective measures alongside self-reports to increase the robustness of the data.

In this study, we examined the impact of cognitive behavioral therapy (CBT) on the quality of life and self-esteem among women diagnosed with colorectal cancer. Our findings indicate that participants who underwent CBT experienced significant improvements in both quality of life and self-esteem compared to the control group. This aligns with previous research highlighting the efficacy of CBT in enhancing psychological well-being among cancer patients [Author et al., Year]. Notably, the observed improvements were more pronounced in cognitive and emotional domains, suggesting that CBT may be particularly effective in addressing these aspects. However, physical functioning improvements were less marked, potentially due to disease progression and treatment-related side effects. These findings are consistent with [Author et al., Year], who reported similar outcomes in a comparable cohort. Unexpectedly, social functioning did not show significant improvement, which may be attributed to the relatively short duration of the intervention. This study’s limitations include its retrospective design and the absence of a standardized measure for disease severity, which could have provided deeper insights into the observed outcomes. Future research should consider longitudinal studies with larger sample sizes and incorporate disease severity indices to further elucidate CBT’s impact across different cancer stages.

To improve the generalizability of future research, it is recommended to include more diverse participant groups, encompassing men, individuals with varying cancer diagnoses, and those from different sociocultural and economic backgrounds. Multi-center studies across various geographic regions could further validate the intervention’s efficacy in broader clinical settings.

### 4.2. Clinical Implications

The findings from this study suggest important clinical implications for the care of cancer patients. Specifically, integrating CBT as a complementary therapy into oncology settings could greatly enhance the psychological well-being of patients, improving both their quality of life and self-esteem. Given the significant improvements in emotional and cognitive functioning observed in the CBT group, incorporating psychosocial interventions should be considered an essential component of comprehensive cancer care. These interventions could be tailored to address the unique psychological challenges faced by patients at different stages of treatment.

Specifically, we emphasize that integrating a self-esteem enhancement intervention into standard cognitive behavioral therapy (CBT) offers a novel approach to improving quality of life for CRC patients. Implementing self-led, virtual reality-based CBT interventions aims to improve sick role adaptation among working-age CRC patients. This innovative method leverages technology to provide immersive therapy experiences, potentially enhancing engagement and outcomes. Combining face-to-face CBT sessions with online self-management activities and telephone consultations has been explored to reduce psychological distress in CRC survivors. This blended approach allows for personalized care and increased accessibility.

### 4.3. Proposals for Future Research

Future research could address several limitations of this study. First, a larger, more diverse sample would increase the generalizability of the findings and allow for subgroup analyses to determine whether the effects of CBT differ across demographic factors such as age, gender, and socioeconomic status. Additionally, longer-term follow-up assessments would be valuable to assess the durability of the intervention’s effects on psychological well-being. Lastly, exploring the combination of CBT with other interventions, such as physical rehabilitation or pharmacotherapy, could provide a more comprehensive approach to supporting cancer patients during chemotherapy.

Future research should address several important questions. First, larger, more diverse samples should be included to assess the generalizability of these findings to men and patients with different types of cancer. Additionally, long-term follow-up studies are needed to determine whether the observed improvements in quality of life and self-esteem are sustained over time. Comparative studies examining the efficacy of CBT relative to other psychosocial interventions (e.g., mindfulness-based stress reduction) could help identify the most effective treatments for enhancing the psychological well-being of cancer patients. Research should also explore whether the benefits of CBT extend to patients with different cancer diagnoses. Finally, investigating the psychological mechanisms underlying CBT’s effects—such as coping strategies or resilience—and examining individual differences that might influence treatment outcomes would provide valuable insights for future intervention development.

## 5. Conclusions

The findings of this study underscore the value of psychotherapy, particularly cognitive behavioral therapy (CBT), as a crucial complement to standard oncology treatment. CBT is especially beneficial during critical periods, such as waiting for medical interventions or when causal treatment is not feasible. By alleviating the intensity of both somatic and psychological symptoms, CBT enhances patients’ quality of life and may contribute to reducing medication use, thereby lowering the associated pharmacological costs.

A key feature of CBT is the active involvement of patients in the therapeutic process. This engagement fosters a heightened sense of personal worth and control over the disease, promoting better emotional resilience and functional adaptation. These advantages position CBT as an invaluable psychological intervention to support oncology patients in managing the physical and emotional challenges of their condition.

Given its proven benefits, integrating CBT programs within oncology centers is strongly recommended. Physicians should actively inform patients about the availability and effectiveness of such interventions, even when psychological symptoms are not immediately apparent. Expanding access to psychotherapy not only enhances patients’ quality of life but also advances a more holistic and patient-centered approach to cancer care.

## Figures and Tables

**Table 1:** Demographic characteristics of the studied sample.

Characteristic	CBT (*n* = 35)	Control (*n* = 32)	Total (*n* = 67)
Marital status			
Single	3 (8.6%)	2 (6.3%)	5 (7%)
Married/Living Together	24 (68.5%)	24 (74.9%)	48 (71.6%)
Widow	3 (8.6%)	4 (12.3%)	7 (10.4%)
Divorced	5 (14.3%)	2 (6.3%)	7 (10.4%)
Education level			
Elementary	2 (5.7%)	1 (3.1%)	3 (4.5%)
Vocational	13 (37.1%)	20 (62.5%)	33 (49.3%)
Middle	13 (37.1%)	9 (28.1%)	22 (32.8%)
High	7 (20.1%)	2 (6.3%)	9 (13.4%)
Total	35 (100.0%)	32 (100.0%)	67 (100.0%)
Age			
Mean	53.31	52.56	52.96
SD	7.51	6.53	7.02
General functioning			
Initial	57.58	57.29	57.43
Final	64.76	54.86	59.81
F = 6.33 *; df = 1;65			
General self-esteem			
Initial	30.80	32.31	31.55
Final	27.06	32.91	29.98
F = 4.46 *; df = 1;65			

SD = standard deviation, CBT = effects of cognitive behavioral therapy, N = number of patients, F = ANOVA coefficient, df = degrees of freedom, * = statistically significant at *p* < 0.05.

**Table 2:** Within-group comparisons of actual and ideal self-averages between baseline (initial) and final assessments.

Group	Self-Esteem			
	Real Self		Ideal Self	
	Initial	Final	Initial	Final
CBT (N = 35)	88.23	92.60	119.03	119.66
t (df = 34)	–3.74 ***		NS	
Control (N = 32)	88.63	86.69	120.94	119.59
t (df = 31)	2.16 *		t = 3.05 **	

CBT = effects of cognitive behavioral therapy, N = number of patient, t = t Student coefficient, df = degrees of freedom, * = statistically significant at *p* < 0.05, ** = statistically significant at *p* < 0.01, *** = statistically significant at *p* < 0.001.

**Table 3:** Interaction effects of therapy participation and the assessment of emotional functioning.

Groups	Group Assessment of Emotional Functioning			
	QLQ Emotional Level		Self-Esteem Emotional Traits	
	Inițial	Final	Inițial	Final
CBT	45.95	55.24	7.63	5.89
Control	48.43	46.61	7.22	7.19
N = 67	F = 6.68 *; df = 1;65		F = 8.43 **; df = 1;65	

CBT = effects of cognitive behavioral therapy, N = number of patient, F = ANOVA coefficient, df = degrees of freedom, * *p* < 0.05; ** *p* < 0.01.

**Table 4:** Interaction effects of therapy participation and assessment of cognitive functioning.

Groups	Assessment of Cognitive Functioning			
	QLQ Emotional Level		Self-Esteem Emotional Traits	
	Initial	Final	Initial	Final
CBT	58.10	72.38	6.03	3.21
Control	54.17	51.56	5.28	3.49
N = 67	F = 6.68 *; df = 1;65		F = 8.43 **; df = 1;65	

CBT = effects of cognitive behavioral therapy, N = number of patient, F = ANOVA coefficient, df = degrees of freedom, * = statistically significant at *p* < 0.05, ** = statistically significant at *p* < 0.01.

## Data Availability

Data are contained within the article.

## Supplementary Materials

The following supporting information can be downloaded at: https://www.mdpi.com/article/10.3390/ejihpe15040042/s1, Table S1: Demographic and Clinical Characteristics of Participants by Year of Study (2020–2024); Table S2: Interaction Effects of Therapy Participation and Physical Functioning Assessment.
