# Supplementary material for: Effectiveness of a Self-Esteem Enhancement Intervention Integrated into Standard CBT Protocol for Improving Quality of Life in Patients with Colorectal Cancer
Source: Eur J Investig Health Psychol Educ. 2025 Mar 22;15(4):42. doi: 10.3390/ejihpe15040042 (PMC12026258; doi:10.3390/ejihpe15040042)
Supplement: Supplementary file 1 [file ejihpe-15-00042-s001.zip › ejihpe-3454713-supplementary.pdf]

Supplementary Table S1: Demographic and Clinical Characteristics of Participants by Year of Study (2020–2024)

Parameters		Year of stady									
		2020		2021		2022		2023		2024	
		N	%	N	%	N	%	N	%	N	%
Groups	CBT	2	3.0%	4	6.0%	8	11.9%	8	11.9%	13	19.4%
	Control group	1	1.5%	3	4.5%	7	10.4%	9	13.4%	12	17.9%
Economic status	Low	0	0.0%	1	1.5%	0	0.0%	3	4.5%	8	11.9%
	Middle	1	1.5%	6	9.0%	14	20.9%	14	20.9%	14	20.9%
	High	2	3.0%	0	0.0%	1	1.5%	0	0.0%	3	4.5%
Education	Primary	0	0.0%	2	3.0%	5	7.5%	7	10.4%	9	13.4%
	Middle	0	0.0%	3	4.5%	6	9.0%	5	7.5%	8	11.9%
	High	3	4.5%	2	3.0%	4	6.0%	5	7.5%	8	11.9%
Cancer	Colon	2	3.0%	0	0.0%	10	14.9%	9	13.4%	8	11.9%
	Rectal	0	0.0%	3	4.5%	2	3.0%	3	4.5%	4	6.0%
	Colo-rectal	1	1.5%	4	6.0%	3	4.5%	5	7.5%	13	19.4%
Stage	1	0	0.0%	2	3.0%	6	9.0%	6	9.0%	14	20.9%
	2	2	3.0%	3	4.5%	2	3.0%	4	6.0%	2	3.0%
	3	1	1.5%	1	1.5%	4	6.0%	6	9.0%	2	3.0%
	4	0	0.0%	1	1.5%	3	4.5%	1	1.5%	7	10.4%

CBT=Effects of Cognitive Behavioral Therapy

Table S2. Interaction Effects of Therapy Participation and Physical Functioning Assessment

Groups	Physical functioning assessment							
	QLQ physical level		QLQ symptoms		QLQ health status		Self-esteem physical characteristics	
	Initial	Final	Initial	Final	Initial	Final	Initial	Final
CBT	67.24	63.64	38.73	35.56	50.00	55.71	7.23	7.23
Control	64.79	61.88	42.01	50.26	48.96	47.14	7.72	7.75
N=67	NS		F=22.37***; df=1;65		F=9.16**;df=1;65		NS	

CBT=Effects of Cognitive Behavioral Therapy, N=number of patient, F=ANOVA coefficient, df=degrees of freedom, NS= No statistically significant differences
